# Temperature dependence of long coherence times of oxide charge qubits

**DOI:** 10.1038/s41598-018-21767-2

**Published:** 2018-02-22

**Authors:** A. Dey, S. Yarlagadda

**Affiliations:** CMP Div., Saha Institute of Nuclear physics, 1/AF Salt Lake, Kolkata, 700064 India

## Abstract

The ability to maintain coherence and control in a qubit is a major requirement for quantum computation. We show theoretically that long coherence times can be achieved at easily accessible temperatures (such as boiling point of liquid helium) in small (i.e., ~10 nanometers) charge qubits of oxide double quantum dots when only optical phonons are the source of decoherence. In the regime of strong electron-phonon coupling and in the non-adiabatic region, we employ a duality transformation to make the problem tractable and analyze the dynamics through a non-Markovian quantum master equation. We find that the system decoheres after a long time, despite the fact that no energy is exchanged with the bath. Detuning the dots to a fraction of the optical phonon energy, increasing the electron-phonon coupling, reducing the adiabaticity, or decreasing the temperature enhances the coherence time.

## Introduction

Although semiconductors are the most widely used functional materials for electronic applications so far, nevertheless, semiconductor devices have some limitations: i) the characteristic length scales are sizeable so that further scaling down the existing system size is quite difficult; and ii) only the charge and spin degrees of freedom are utilized. On the other hand, owing to significantly smaller extent of the wavefunction, transition metal oxides can meet the miniaturization demands much better than semiconductors. Furthermore, oxides offer a vastly richer physics involving diverse spin, charge, lattice, and orbital correlations^1–7^. Low-dimensional oxides present new opportunities for devices where these diverse correlations can be optimized by engineering many-body interactions, fields, geometries, disorder, strain, etc. Therefore, oxides may be viewed as one of the best candidates to replace semiconductors in future electronic devices.

Construction of scalable quantum computers has motivated identification of coherent two-level solid-state systems. A simple solid-state two-level system is the charge qubit. Charge qubit holds promise for high-speed manipulation due to strong coupling of the electron to electric field. On the other hand, large coherence times have not been achieved so far in the semiconductor double quantum dot (DQD) systems studied as charge qubits^8–18^. Decoherence, due to system-environment interactions, degrades the precious resource of quantum mechanical superpositions^19,20^ and is one of the main obstacles in quantum information processing. The temperature of the environment affects the reduced system dynamics and introduces additional relaxation channel for the system. Furthermore, the semiconductor quantum dots employed in the decoherence studies had a relatively large diameter (i.e., ~200 nm) with the corresponding electron temperatures being low (i.e., ~100 mK). Thus concomitant realization of fast operation and large coherence times in small solid state qubits at easily accessible temperatures (such as boiling points of liquid helium and liquid nitrogen and room temperature), although very useful for quantum computation, has been elusive so far.

Here we show that, compared to a semiconductor DQD, an oxide DQD (modeled with phonons as the only source of decoherence) may yield significantly longer coherence times (i.e., longer by at least an order of magnitude) at higher temperatures (i.e., higher by an order of magnitude) and in smaller sized systems (i.e., smaller by an order of magnitude). Thus we illustrate the device potential of low-dimensional oxides through our analysis of a manganite-based DQD as a charge qubit. We employ a duality transformation, that is potentially widely applicable, to map a strong-coupling electron-phonon problem to a weak-coupling problem (where the small parameter is the inverse of that in the strong-coupling case); we consider a system that is initially decoupled from the phonon bath in the polaronic frame of reference and solve the non-Markovian quantum master equation.

## Formulation

### DQD model with environment

We consider a laterally coupled DQD system for our two-level qubit. The charge in the DQD system is denoted (*N*_1_, *N*_2_) with *N*_1_ and *N*_2_ being the number of electrons on dots 1 and 2, respectively. The quantum dots are taken to be identical with the same charging energy *E*_*C*_ = *e*^2^/*C* where *e* is the charge of an electron and *C* is the capacitance between the dot and its surroundings. The capacitance *C* can be conservatively approximated by the self-capacitance *C*_0_ = 4*ε*_*m*_*ε*_0_*D*^21^ which for a manganite dot with dielectric constant *ε*_*m*_ = 10 and diameter *D* = 10 nm yields *E*_*C*_ ~ 0.05 eV. We analyze situations where the thermal energy *k*_*B*_*T* as well as the the detuning Δ*ε* ≡ *ε*_1_ − *ε*_2_ (between the lowest energy levels in the two dots) are both smaller than *E*_*C*_ so that the dynamics of a single electron can be studied when |*N*_1_ − *N*_2_| = 1. Consequently, we define the relevant charge states as |10〉 ≡ (*N* + 1, *N*) and |01〉 ≡ (*N*, *N* + 1).

The coupled dots are described by the following Hamiltonian of a single electron tunneling between them:

1 $${H}_{{\rm{DQD}}}={\varepsilon }_{1}{m}_{1}+{\varepsilon }_{2}{m}_{2}-\frac{{J}_{\perp }}{2}({c}_{1}^{\dagger }{c}_{2}+{c}_{2}^{\dagger }{c}_{1})+{J}_{\parallel }{m}_{1}{m}_{2},$$

where the electron destruction operator in dot *i* is defined as *c*_*i*_ and ${m}_{i}\equiv {c}_{i}^{\dagger }{c}_{i}$. Furthermore, the energies *ε*_*i*_ and the interdot tunnel coupling $\frac{{J}_{\perp }}{2}$ are adjusted by external gates; the nearest neighbor repulsion ${J}_{\parallel }$ is due to Coulomb interaction. ${J}_{\parallel }$*m*_1_*m*_2_ is included for generality although its value is zero here. The total Hamiltonian is expressed as *H* = *H*_DQD_ + *H*_P_ + *H*_EP_ where the additional term ${H}_{{\rm{P}}}={\sum }_{i,k}\,{\omega }_{k}{a}_{i,k}^{\dagger }{a}_{i,k}$ is due to the optical phonon environment while ${H}_{{\rm{EP}}}=\frac{1}{\sqrt{N}}\,{\sum }_{i,k}\,{g}_{k}{\omega }_{k}({m}_{i}-\frac{1}{2})\,({a}_{i,k}+{a}_{i,k}^{\dagger })$ is due to the electron-phonon interaction; here, *a*_*j*,*k*_ is the destruction operator of mode k phonons at site j, *g*_*k*_ is the electron-phonon coupling strength, and *ω*_*k*_ is the optical phonon frequency with weak dispersion. The role played by the acoustic phonons in decoherence will be presented in the discussion section.

In the strong coupling regime, to perform perturbation theory effectively, we locally displace the harmonic oscillators by Lang-Firsov (LF) transformation^22^ *H*^*L*^ ≡ *e*^*S*^ *He*^−*S*^ with $S=-\frac{1}{\sqrt{N}}\,{\sum }_{i,k}\,{g}_{k}({m}_{i}-\frac{1}{2})\,({a}_{i,k}-{a}_{i,k}^{\dagger })$. In the LF/polaronic frame, the electron is clothed with phonons reducing the tunneling term *J*_⊥_ in Eq. (1) to ${J}_{\perp }^{{\rm{mf}}}\equiv {J}_{\perp }{e}^{-\frac{1}{N}{\sum }_{k}{g}_{k}^{2}\coth \frac{\beta {\omega }_{k}}{2}}$. This reduction of the polaronic tunneling at enhanced temperatures occurs for the same reason as that in a polaron band. Therefore, in the DQD, the single particle energy (~${J}_{\perp }^{{\rm{mf}}}$) is much smaller than the charging energy *E*_*C*_. The redefined polaronic system, the bath environment with displaced harmonic oscillators, and the interaction term in the LF/polaronic frame are respectively given by

2 $${H}_{s}^{L}={\varepsilon }_{1}{m}_{1}+{\varepsilon }_{2}{m}_{2}-\frac{{J}_{\perp }^{{\rm{mf}}}}{2}({c}_{1}^{\dagger }{c}_{2}+{c}_{2}^{\dagger }{c}_{1})+{J}_{\parallel }{m}_{1}{m}_{2},$$

3 $${H}_{R}^{L}=\sum _{i,k}\,{\omega }_{k}{a}_{i,k}^{\dagger }{a}_{i,k},$$

and

4 $${H}_{I}^{L}=-\frac{1}{2}[{J}_{\perp }^{+}{c}_{1}^{\dagger }{c}_{2}+{J}_{\perp }^{-}{c}_{2}^{\dagger }{c}_{1}],$$

where the fluctuation of the local phonons around the mean phonon field $${J}_{\perp }^{{\rm{mf}}}$$ is given by $${J}_{\perp }^{\pm }=$$$${J}_{\perp }{e}^{\pm \frac{1}{\sqrt{N}}{\sum }_{k}{g}_{k}[({a}_{\mathrm{2,}k}-{a}_{\mathrm{2,}k}^{\dagger })-({a}_{\mathrm{1,}k}-{a}_{\mathrm{1,}k}^{\dagger })]}-{J}_{\perp }^{{\rm{mf}}}$$; additionally, the destruction operators *c*_*i*_ and *a*_*i*,*k*_ correspond to fermions and phonons and operate in the LF frame. In the polaronic frame of reference, the Hamiltonian is given as the sum of an unperturbed part ($${H}_{s}^{L}+{H}_{R}^{L}$$) and a weak perturbation ($${H}_{I}^{L}$$). Although electron-phonon interaction is strong in the laboratory frame, interaction in the polaronic frame (where the interaction is merely between the fermion and local phonon fluctuations) is weak and therefore suits a perturbative treatment. The small parameter of the perturbation theory in the polaronic frame of reference is ~$${J}_{\perp }/(g{\omega }_{u})$$ with coupling strength *g* and optical phonon frequency *ω*_*u*_ defined in Eq. (10); a detailed analysis of the small parameter in polaronic frame is given in Appendix B of ref.^23^. On the other hand, in the laboratory frame of reference, the perturbation parameter is ~$$(g{\omega }_{u})/{J}_{\perp }$$ [for details of the small parameter in the laboratory frame, see ref.^24^]. Thus the polaronic (LF) transformation is actually a duality transformation that maps the original strong-coupling problem in the laboratory frame to a weak-coupling problem in the polaronic frame.

### Polaron dynamics

The dynamics of the system is described in terms of the reduced density matrix of the system *ρ*_*s*_(*t*) ≡ Tr_*R*_[*ρ*_*T*_(*t*)] where the degrees of freedom of the bath are traced out from the total system-environment density matrix *ρ*_*T*_(*t*). We start with the simply separable initial state *ρ*_*T*_(0) = *ρ*_*s*_(0) ⊗ *R*_0_ in the polaronic frame of reference with the expectation that perturbation at large coupling will not produce much change to the state of the system^23^. Here, *R*_0_ is the phonon density matrix at thermal equilibrium given by ${R}_{0}={\sum }_{\{{n}_{k}\}}\,|\{{n}_{k}\}{\rangle }_{ph}\,{}_{ph}\langle \{{n}_{k}\}|{e}^{-\beta {\bar{\omega }}_{n}}/Z$; the phonon eigenstate and eigenenergy are given by $|\{{n}_{k}\}{\rangle }_{ph}\equiv |\{{n}_{1}^{k}\},\{{n}_{2}^{k}\}{\rangle }_{ph}$ and ${\bar{\omega }}_{n}\equiv {\sum }_{k}\,{\omega }_{k}({n}_{1}^{k}+{n}_{2}^{k})$ with ${n}_{1}^{k}$ and ${n}_{2}^{k}$ being the mode k phonon occupation numbers in dots 1 and 2. This separable initial state can be obtained in a physical system such as an oxide-based DQD by using a small value of *J*_⊥_/*ω*_*k*_^25^.

We analyze the reduced dynamics of the system by the second-order, time-convolutionless, non-Markovian, quantum-master equation in the interaction picture [i.e., Redfield equation (see ref.^26^)]:

5 $$\frac{d{\tilde{\rho }}_{s}(t)}{dt}=-{\int }_{0}^{t}\,d\tau T{r}_{R}[{\tilde{H}}_{I}^{L}(t),[{\tilde{H}}_{I}^{L}(\tau ),{\tilde{\rho }}_{s}(t)\otimes {R}_{0}]].$$

Here, an operator *A* is expressed in the interaction picture representation as $\tilde{A}(t)={e}^{i({H}_{s}^{L}+{H}_{R}^{L})t}A{e}^{-i({H}_{s}^{L}+{H}_{R}^{L})t}$. For our analysis we use the eigenstate basis $\{|{\varepsilon }_{s}\rangle =\frac{\mathrm{|10}\rangle -\mathrm{|01}\rangle }{\sqrt{2}},\,|{\varepsilon }_{t}\rangle =\frac{\mathrm{|10}\rangle +\mathrm{|01}\rangle }{\sqrt{2}}\}$ (with eigenenergies *ε*_*s*_ and *ε*_*t*_) for zero detuning (Δ*ε* = 0) and the basis {|10〉, |01〉} for strong detuning (${\rm{\Delta }}\varepsilon \gg {J}_{\perp }^{{\rm{mf}}}$). For the zero (finite) detuning case, to analyze coherence and population, we solve for the offdiagonal density matrix element ${\tilde{c}}_{st}(t)\equiv \langle {\varepsilon }_{s}|{\tilde{\rho }}_{s}(t)|{\varepsilon }_{t}\rangle$ $({\tilde{c}}_{10}(t)\equiv \langle \mathrm{10|}{\tilde{\rho }}_{s}(t\mathrm{)|01}\rangle )$ and the diagonal element ${\tilde{p}}_{s}(t)\equiv \langle {\varepsilon }_{s}|{\tilde{\rho }}_{s}(t)|{\varepsilon }_{s}\rangle$ $({\tilde{p}}_{10}(t)\equiv \langle \mathrm{10|}{\tilde{\rho }}_{s}(t\mathrm{)|10}\rangle )$.

### Zero detuning

For the case when Δ*ε* = 0, from Eq. (5), we get the following equations of motion for the offdiagonal and diagonal elements of ${\tilde{\rho }}_{s}(t)$.

6 $$\begin{array}{rcl}{\dot{\tilde{c}}}_{st}(t) & = & -[{\tilde{c}}_{st}(t)\,\sum _{\bar{n}+\bar{m}={\rm{even}},0}\,{{\mathscr{J}}}_{nm}\,{f}_{nm}(t)\\  &  & -\frac{i{e}^{i\delta \varepsilon t}}{4}({\tilde{c}}_{st}(t){e}^{-i\delta \varepsilon t}\sum _{\bar{n}+\bar{m}={\rm{odd}}}\,{{\mathscr{J}}}_{nm}\{{{\mathbb{F}}}_{nm}^{+}(t)-{{\mathbb{F}}}_{nm}^{-\ast }(t)\}-{\rm{H}}.{\rm{c}}.)],\end{array}$$

and

7 $$\begin{array}{rcl}{\dot{\tilde{p}}}_{s}(t) & = & -\frac{1}{4i}\,\sum _{\bar{n}+\bar{m}={\rm{odd}}}\,{{\mathscr{J}}}_{nm}[{\tilde{p}}_{s}(t)\{({{\mathbb{F}}}_{nm}^{+}(t)+{{\mathbb{F}}}_{nm}^{-}(t))\\  &  & -{\rm{H}}.{\rm{c}}\mathrm{.\}}-\{{{\mathbb{F}}}_{nm}^{-}(t)-{\rm{H}}.{\rm{c}}\mathrm{.\}}],\end{array}$$

where, $\bar{n}\equiv {\sum }_{k}\,({n}_{1}^{k}+{n}_{2}^{k})$, $\bar{m}\equiv {\sum }_{k}\,({m}_{1}^{k}+{m}_{2}^{k})$, ${{\mathscr{J}}}_{nm}={{(}_{ph}{\langle \{{n}_{k}\}|{J}_{\perp }^{+}|\{{m}_{k}\}\rangle }_{ph})}^{2}\frac{{e}^{-\beta {\bar{\omega }}_{n}}}{Z}$, ${f}_{nm}(t)=\frac{\sin ({\bar{\omega }}_{n}-{\bar{\omega }}_{m})t}{{\bar{\omega }}_{n}-{\bar{\omega }}_{m}}$, and ${{\mathbb{F}}}_{nm}^{\pm }(t)=\frac{{e}^{i({\bar{\omega }}_{n}-{\bar{\omega }}_{m}\pm \delta \varepsilon )t}-1}{{\bar{\omega }}_{n}-{\bar{\omega }}_{m}\pm \delta \varepsilon }$ with *δε* ≡ *ε*_*s*_ − *ε*_*t*_. To understand coherence and population evolution, we define the coherence factor C_st_(*t*) ≡ |*c*_*st*_(*t*)|/|*c*_*st*_(0)| which can be obtained by solving Eq. (6) and its complex conjugate; we also calculate the population difference P_st_(*t*) ≡ (2*p*_*s*_(*t*) − 1)/(2*p*_*s*_(0) − 1).

### Finite detuning

As a strategy to mitigate decoherence, we employ sizeable energy detuning. For the case of finite detuning ${\rm{\Delta }}\varepsilon \gg \delta \varepsilon$, the equations for the offdiagonal and diagonal density matrix elements [obtained from Eq. (5)] are given by

8 $$\begin{array}{rcl}{\dot{\tilde{c}}}_{10}(t) & = & -\frac{i}{4}\,\sum _{\{{n}_{k}\},\{{m}_{k}\}}\,{{\mathscr{J}}}_{nm}[{\tilde{c}}_{10}(t)\,({ {\mathcal F} }_{nm}^{-\ast }(t)-{ {\mathcal F} }_{nm}^{+}(t))\\  &  & +{(-\mathrm{1)}}^{(\bar{n}+\bar{m})}{e}^{2i{\rm{\Delta }}\varepsilon t}\times {\tilde{c}}_{10}^{\ast }(t)\,({ {\mathcal F} }_{nm}^{-}(t)-{ {\mathcal F} }_{nm}^{+\ast }(t))],\end{array}$$

and

9 $$\begin{array}{rcl}{\dot{\tilde{p}}}_{10}(t) & = & -\frac{1}{4i}\,\sum _{\{{n}_{k}\},\{{m}_{k}\}}\,{{\mathscr{J}}}_{nm}[{\tilde{p}}_{10}(t)\{({ {\mathcal F} }_{nm}^{+}(t)+{ {\mathcal F} }_{nm}^{-}(t))\\  &  & -{\rm{H}}.{\rm{c}}\mathrm{.\}}-\{{ {\mathcal F} }_{nm}^{-}(t)-{\rm{H}}.{\rm{c}}.\}],\end{array}$$

where ${ {\mathcal F} }_{nm}^{\pm }(t)=\frac{{e}^{i({\bar{\omega }}_{n}-{\bar{\omega }}_{m}\pm {\rm{\Delta }}\varepsilon )t}-1}{{\bar{\omega }}_{n}-{\bar{\omega }}_{m}\pm {\rm{\Delta }}\varepsilon }$. To characterize the dynamics, we define the relevant coherence factor C_10_(*t*) ≡ |*c*_10_(*t*)|/|*c*_10_(0)| and the population difference P_10_(*t*) ≡ (2*p*_10_(*t*) − 1)/(2*p*_10_(0) − 1) which can be calculated from the above two equations. Furthermore, when both Δ*ε* and *δε* are non-negligible, a general derivation of the matrix elements of the four terms on the right-hand side of Eq. (5) is given in the supplementary information.

## Results and Analysis

In oxides such as the manganites, we approximate the density of states D(*ω*_*k*_) of our multimode baths by a generalization of the Einstein model and take it to be a box function of small width *ω*_*u*_ − *ω*_*l*_ (=0.1*ω*_*u*_) and height $\frac{1}{({\omega }_{u}-{\omega }_{l})}$

10 $${\rm{D}}({\omega }_{k}){g}_{k}^{2}={g}^{2}\frac{N}{{\omega }_{u}-{\omega }_{l}}{\rm{\Theta }}({\omega }_{k}-{\omega }_{l})\,{\rm{\Theta }}({\omega }_{u}-{\omega }_{k}),$$

where Θ(*ω*) is the unit step function. Notice that $\frac{1}{N}\,{\sum }_{k}\,{g}_{k}^{2}={g}^{2}$.

In our calculations, we have employed experimentally realistic values of parameters in perovskite manganites. For tunneling we chose *J*_⊥_/*ω*_*u*_ = 0.5 & 2.8 with phonon energy *ħω*_*u*_ = 0.05 eV; these values of *J*_⊥_ can be achieved by adjusting a gate voltage. There is compelling evidence of strong electron-phonon coupling in manganites^27,28^. As regards strong couplings, we used *g* = 2.0 & 2.5. The strength of electron-phonon coupling can be varied by using different rare earth (RE) elements (such as *La*, *Pr*, & *Nd*) in the oxide *RE*_1−*x*_*Ca*_*x*_*MnO*_3_^29^. Thus, we can study decoherence for a reasonable range of small parameter values ${J}_{\perp }/\mathrm{(2}\sqrt{2}g{\omega }_{u})$^23^. Furthermore, using manganites around the ferromagnetic colossal magnetoresistive regime would aid controllability in the DQDs.

Deviations from exponential decay have been predicted [see ref.^30^] and observed [see ref.^31^] at short times for unstable quantum states. In fact, similar to ref.^31^, our long-time behavior of coherence is also given exp(−*α* − *t*/*τ*) where *τ* is the coherence time and *α* > 0 is a constant much smaller than unity.

**Figure 1:**
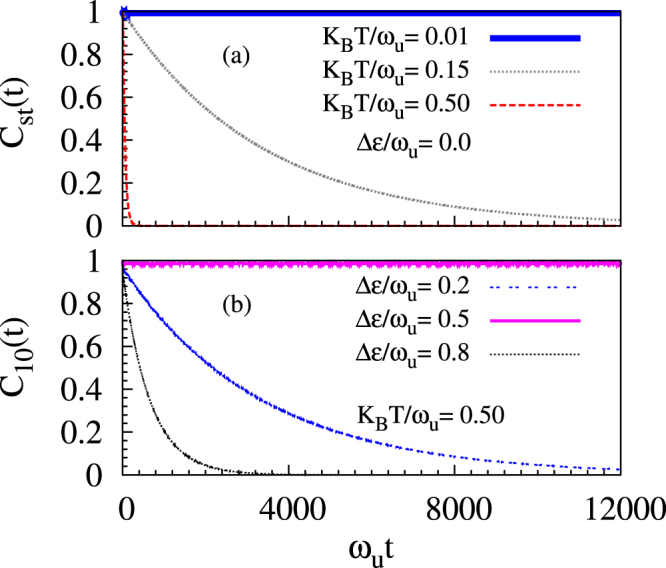
Exponential decay of coherence as a function of dimensionless time *ω*_*u*_*t* plotted for (**a**) detuning energy Δ*ε* = 0.0 (where largest decoherence is expected) and at values of dimensionless thermal energy K_B_T/*ω*_*u*_ = 0.01 (near boiling point of liquid helium for *ħω*_*u*_ = 0.05 eV), =0.15 (near boiling point of liquid nitrogen), and =0.5 (near room temperature); for (**b**) dimensionless thermal energy K_B_T/*ω*_*u*_ = 0.5 at various detuning energies Δ*ε*/*ω*_*u*_ reflecting different probabilities for resonance. All the plots are at values of adiabaticity *J*_⊥_/*ω*_*u*_ = 0.5 and electron-phonon coupling *g* = 2 that are realizable in oxide DQDs.

**Figure 2:**
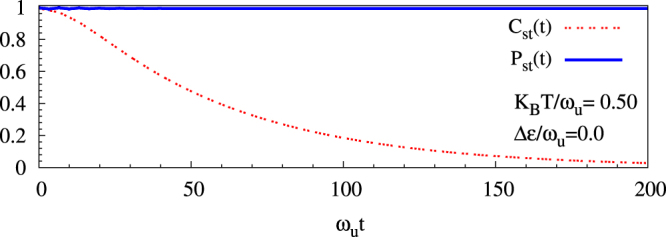
Time dependence, of coherence and population difference, depicting exponential decay of coherence while population remains unchanged for the case of maximum decoherence (i.e., detuning Δ*ε* = 0) at around room temperature (i.e., K_B_T/*ω*_*u*_ = 0.5 for *ħω*_*u*_ = 0.05 eV). Adiabaticity *J*_⊥_/*ω*_*u*_ = 0.5 and electron-phonon coupling *g* = 2 in the plot.

In Fig. 1(a) we investigate nature of decoherence for zero detuning and various temperatures. At a very low temperature K_B_T = 0.01*ω*_*u*_ (i.e., around boiling point of liquid helium for *ħω*_*u*_ = 0.05 eV), we see that coherence does not decay; whereas, with increasing temperature it decays more rapidly. We compared numerical values of coherence for temperatures 0.01*ω*_*u*_, with those at much lower temperatures including 0 K. Over the entire time range in Fig. 1, with respect to the zero temperature case, we find that the coherence values for K_B_T = 0.01*ω*_*u*_ do not change at least up to the twelfth decimal place. This leads us to infer that *τ* > 100 s at these low temperatures (see Table 1). Similarly, at finite detuning values as well, by contrasting coherence at temperature 0.01*ω*_*u*_ with those at much lower temperatures, we report large coherence time *τ* > 100 s in Table 1. With increasing temperatures, not only do excited phonon states appear with enhanced thermal probability but also the number of degenerate phonon eigenstates increases. Even if the total phonon bath does not exchange excitation with the system (since $\delta \varepsilon \ll {\omega }_{u}$), this leads to a fluctuation in local phonon excitations causing destruction of coherence; consequently, there is a decay in coherence while the population difference remains unchanged as shown in Fig. 2. Since there is no exchange of energy between the bath and the polaron, other unoccupied single particle states will not be relevant in producing decoherence. The term *f*_*nm*_(*t*) in Eq. (6) represents the contributions from degenerate excited phonon eigenstates. At temperatures ${{\rm{K}}}_{{\rm{B}}}{\rm{T}}\ll {\omega }_{u}$, the phonon ground state (although being probabilistically dominant) produces no decoherence as the strength of decoherence ${{\mathscr{J}}}_{00}=0$; furthermore, the next dominant term is proportional to ~exp(−*βω*_*u*_) becomes non-negligible only at much higher temperatures (i.e., K_B_T/*ω*_*u*_ = 0.15 & 0.5).

**Table 1:** Coherence times at various values of scaled thermal energy K_B_T/*ω*_*u*_ and detuning energy Δ*ε*/*ω*_*u*_.

Δ*ε*/*ω*_*u*_	0.0	0.2	0.5	0.8
K_B_T/*ω*_*u*_				
0.01	>100 s	>100 s	>100 s	>100 s
0.15	50 ps	24 *μ*s	0.1 s	83 ns
0.50	1 ps	47 ps	0.75 *μ*s	10 ps

We will now comment on the contribution of the vacuum state of the bath to decoherence at low temperatures. The polaronic transformation produces composite fermions (involving fermions clothed with phonons) and displaces the simple harmonic oscillators by changing the vacuum of the bath. In fact, the Hamiltonian ${H}_{R}^{L}$ corresponds to displaced harmonic oscillators; the displacement depends on the interaction strength as well as on the electronic occupation state. The off-diagonal elements of the system density matrix in the laboratory and the polaronic frame are proportional to each other [see analysis for oxide DQD in Eqs (81)–(84) in Sec. VIII of ref.^25^]. When the vacuum state of the bath becomes a coherent state, the analysis in ref.^25^ shows that we get very small decoherence. At strong nonadiabaticity, the lattice distortion quickly follows the location of the electron resulting in a coherent dynamics of the polaron [see section VIII of ref.^25^].

Next, we will contrast the above picture of coherence in a small oxide DQD with that in a bulk material governed by the Holstein model^32^. As the temperature is varied, two qualitatively different mechanisms are relevant for transport in a bulk system of electrons strongly coupled to optical phonons, namely, the coherent band-like motion and the incoherent random hopping of small polarons. At higher temperatures, the overlap between the simple-harmonic-oscillator wave functions of host molecules on neighboring sites decreases because higher eigenfunctions with more nodes come into play; consequently, the polaron bandwidth decreases. At higher temperatures, the random process dominates over the band motion; the crossover from band-like motion to hopping conduction occurs when the uncertainty in energy (produced by electron-phonon scattering) is comparable to half the bandwidth^33,34^.

To obtain long coherence times even at elevated temperatures, we introduce detuning in the DQD. At nonzero temperatures, excited phonon states come into the picture. Even for a fixed phonon eigenenergy, there may be degeneracy in terms of different phonon occupation arrangements at dots 1 and 2. This results in fluctuations in local phonon fields (even in the case of a single excited phonon) leading to decoherence. Such a contribution is taken into account by the first term in Eq. (6). Now, a finite detuning counters such fluctuations [compare Eqs (6) and (8)]; unlike the zero detuning situation, finite detuning makes decoherence condition more difficult in terms of energy matching conditions.

For the situation where Δ*ε* equals any of the phonon eigenenergy differences ${\bar{\omega }}_{n}-{\bar{\omega }}_{m}$, there is a decay of coherence due to resonance. Decoherence for a particular detuning depends on the thermal probability of the phonon excitation that produces the resonance condition ${\bar{\omega }}_{n}-{\bar{\omega }}_{m}={\rm{\Delta }}\varepsilon$ [see Eq. (8)]. We plot the coherence factor in Fig. 1(b) at around the room temperature (i.e., K_B_T = 0.5*ω*_*u*_ for *ħω*_*u*_ = 0.05 eV). Here, we see that the coherence time is much longer for Δ*ε*/*ω*_*u*_ = 0.5 compared to the other two cases Δ*ε*/*ω*_*u*_ = 0.2 & 0.8. Now, when 0.1*ω*_*u*_ < Δ*ε* ≤ 0.2*ω*_*u*_, since 2(*ω*_*u*_ − *ω*_*l*_) = 0.2*ω*_*u*_, resonance condition ${\bar{\omega }}_{n}-{\bar{\omega }}_{m}={\rm{\Delta }}\varepsilon$ can be met by two-phonon excitation with a thermal probability ~$\exp (-2\beta {\omega }_{u})$ as can be seen from Eq. (8). Similarly, when 0.5*ω*_*u*_ ≤ Δ*ε* < 0.6*ω*_*u*_, since 5*ω*_*l*_ − 4*ω*_*u*_ = 0.5*ω*_*u*_, resonant condition ${\bar{\omega }}_{n}-{\bar{\omega }}_{m}={\rm{\Delta }}\varepsilon$ can be met by four-phonon excitation with the relevant thermal probability being ~$\exp (-4\beta {\omega }_{u})$ (which is smaller comparatively). However, when 0.8*ω*_*u*_ ≤ Δ*ε* < 0.9*ω*_*u*_, since 2*ω*_*l*_ − *ω*_*u*_ = 0.8*ω*_*u*_, resonance is established by single-phonon excitation with a corresponding thermal probability ~$\exp (-\beta {\omega }_{u})$.

**Figure 3:**
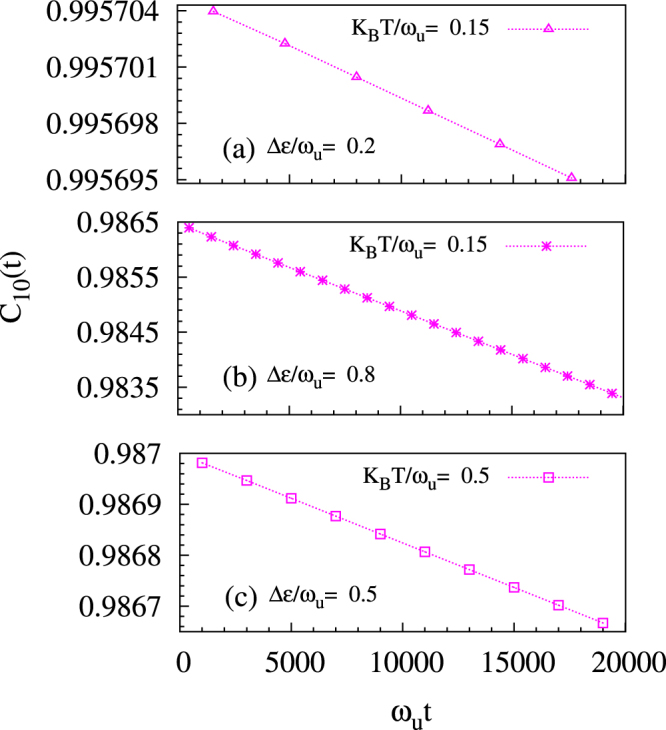
Depiction that exponential decay of coherence is linear at small times. The coherence time is inversely proportional to the slope. The coherence values are averaged over intervals of width between successive points. At a fixed temperature, among the detunings considered, the least decoherence occurs when detuning Δ*ε*/*ω*_*u*_ = 0.5 since it has the lowest chance for resonance. Figures were drawn at adiabaticity *J*_⊥_/*ω*_*u*_ = 0.5 and electron-phonon coupling *g* = 2.

Next, we plot Fig. 3, by exploiting the linearity of the exponential decay at times much smaller than the large coherence times realized near boiling point of liquid nitrogen (i.e., K_B_T = 0.15*ω*_*u*_ for *ħω*_*u*_ = 0.05 eV) at detuning Δ*ε*/*ω*_*u*_ = 0.2 & 0.8 and for K_B_T = 0.5*ω*_*u*_ at Δ*ε*/*ω*_*u*_ = 0.5. Figure 3 is in agreement with Fig. 1. The numerical values of coherence times for the cases in Figs 1 and 3 are reported in Table 1; at temperatures much above boiling point of liquid helium, we see that with a properly chosen detuning one can get a coherence time many orders of magnitude larger than the zero-detuning case.

**Figure 4:**
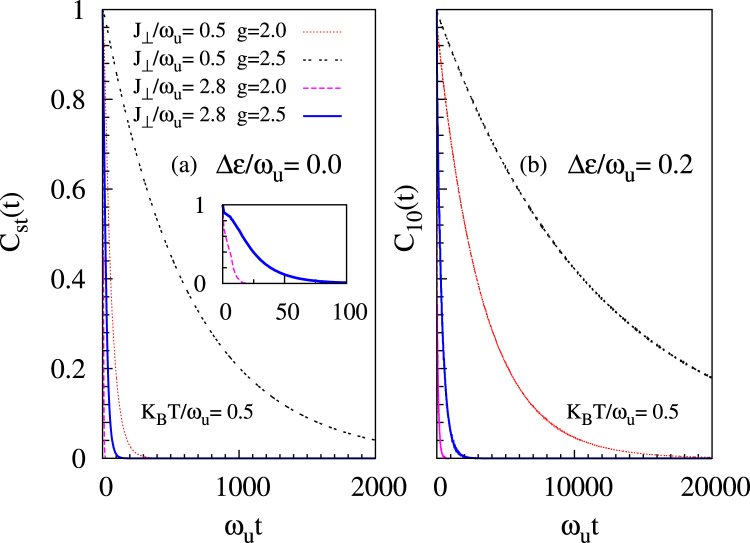
Plot showing coherence decays faster (slower) with increasing adiabaticity *J*_⊥_/*ω*_*u*_ (electron-phonon coupling *g*) at two representative values of detuning, i.e., Δ*ε*/*ω*_*u*_ = 0.0 & 0.2, and near room temperature.

Lastly, in Fig. 4, we plot the coherence factor for different values of the coupling *g* and the tunneling amplitude *J*_⊥_. For a fixed tunneling, coherence is maintained for a longer time when the electron-phonon coupling is stronger. On the other hand, at a fixed value of the coupling, decoherence is enhanced when tunneling increases. These results are consistent with the fact that decoherence diminishes at lower values of the small parameter $\frac{{J}_{\perp }}{2\sqrt{2}g\omega }$^23,25^. Comparing Fig. 4(a,b), we again see that finite-detuning provides longer coherence times.

We will now comment on the nature of the steady state. The steady state, attained after the long coherence time, corresponds to decay of off-diagonal terms. Now, at zero detuning, the population difference hardly changes because no exchange of energy can take place between the bath and the fermion [see Fig. 2 of the present paper and Fig. 8 in ref.^25^]. On the other hand, at finite detuning, the population difference can also decay [see Fig. 11 in ref.^23^]. Thus, the steady state is always uniquely defined.

Next, we will compare our approach with other earlier treatments. In an interesting fundamental study of single-polaron dynamics in a Holstein model (in a non-extreme parameter regime similar to ours)^35^, it was demonstrated that, at strong coupling and for weak external driving electric field, the system experiences essentially undamped Bloch oscillations, thereby supporting our results. In addition, nonadiabatic transition has an exponentially small probability; we expect this picture to hold for ${k}_{B}T\ll {\omega }_{u}$ (~ polaron excitation gap). Next, for the initial condition where the particle is uncoupled to the phonons in the laboratory frame, studies of dynamics in a Holstein model at strong couplings can be found in refs^36,37^.

## Discussion

We will now lend some perspective to the approximations made in this paper. We approximated a quantum dot as a single electronic site coupled to a multimode bath of optical phonons where the density of states is treated as a generalization of the Einstein model. We employed a second-order, time-convolutionless, non-Markovian, quantum-master equation (i.e., Redfield equation) to study the dynamics. The accuracy of the above approximations needs to be studied by alternate approaches. As regards considerations pertaining to non-extreme parameter regime, the small parameter will not be very small when the gate voltage in our manganite-based DQD is adjusted to keep the tunneling term in the adiabatic regime such as in bulk manganites; then, the importance of higher order terms in the perturbation theory needs to be investigated.

As pointed in many papers on oxides^38^, the effective Hamiltonian for obtaining the phase diagram is given by:

11 $$H={H}_{{\rm{ke}}}^{{e}_{g}}+{H}_{{\rm{Hund}}}+{H}_{{\rm{inter}}-{\rm{site}}}^{{\rm{AF}}}+{H}_{{\rm{el}}-{\rm{ph}}}^{{e}_{g}}+{H}_{{\rm{el}}-{\rm{el}}}^{{e}_{g}}$$

In the above equation, the electron-phonon coupling term ${H}_{{\rm{el}}-{\rm{ph}}}^{{e}_{g}}$ contains breathing mode and Jahn-Teller modes (i.e., modes corresponding to optical phonons) because they correspond to the dominant electron-phonon interactions. Here, since we deal with a single *e*_*g*_ electron, the electron-electron-interaction term ${H}_{{\rm{el}}-{\rm{el}}}^{{e}_{g}}$ is not relevant. Furthermore, in the ferromagnetic colossal magnetoresistive regime of manganites, the Hund’s coupling *H*_Hund_ and the inter-site antiferromagnetic coupling (due to superexchange) ${H}_{{\rm{inter}}-{\rm{site}}}^{{\rm{AF}}}$ are already taken into account in obtaining the magnetic phase. Thus, we consider only the kinetic term ${H}_{{\rm{ke}}}^{{e}_{g}}$ and ${H}_{{\rm{el}}-{\rm{ph}}}^{{e}_{g}}$. Thus our primary objective is to model the decoherence due to optical phonons. We find that when only optical phonons are the source of decoherence and when the system strongly couples to the optical phonons, the coherence times are long. The other significant sources for decoherence could be the acoustic phonons and charge noise.

When acoustic-phonon wavelength is longer than the size of the dot, the electron-phonon coupling is inefficient, since the entire dot potential is just shifted uniformly up and down, and no longer couples different orbitals/states (in the dot) to each other. The minimum excitation energy for an acoustic phonon corresponds to the wavevector 2*π*/*D*. The smaller the value of the diameter *D*, the larger the excitation energy. Assuming a simple sinusoidal dispersion^39^ of the form ${\omega }_{{\rm{\max }}}^{{\rm{ac}}}\,\sin (ka\mathrm{/2)}$, for ${\omega }_{{\rm{\max }}}^{{\rm{ac}}}=0.02$ eV and *k* = 2*π*/(25*a*) [when *D* = 10 nm and lattice constant *a* = 0.4 nm], we get the lowest excitation energy ${\omega }_{{\rm{\min }}}^{{\rm{ac}}}$ to be 0.02 sin(*π*/25) eV ≈ 2.5 meV. For *La*_0.7_*Sr*_0.3_*MnO*_3_, the lowest excitation energy is 2 meV [see Fig. 4(e) in ref.^40^]. Now, 2 meV is larger than the tunneling term ${J}_{\perp }{e}^{-{g}^{2}}\sim 0.5$ meV. In fact, the tunneling term can be decreased by adjusting the gate voltage. For larger dots with *D* = 200 nm, such as in semiconductor quantum dots, we get much smaller excitation energy (i.e., ~0.125 meV); consequently, exchange of energy can take place between acoustic phonons and the electron leading to decoherence. On taking acoustic phonons into account for *D* = 10 nm manganite DQD, at the boiling point of liquid helium, ${{\rm{K}}}_{{\rm{B}}}{\rm{T}}/{\omega }_{{\rm{\min }}}^{{\rm{ac}}}\sim 0.36\,{\rm{meV}}\mathrm{/2.5}\,{\rm{meV}}\sim 0.14$; then, based on Table 1, it should be clear that the decoherence times of the second row (i.e., K_B_T/*ω*_*u*_ = 0.15 case) should be achievable at various ${\rm{\Delta }}\varepsilon /{\omega }_{{\rm{\min }}}^{{\rm{ac}}}$ when we adjust ${J}_{\perp }/{\omega }_{{\rm{\min }}}^{{\rm{ac}}}=0.5$ using the gate voltage. Thus, decoherence times up to ~0.1 s can be realized at temperatures elevated significantly above dilution refrigerator temperatures (i.e., at boiling point of liquid helium). Hence, we expect that our claim of long coherence time will not be really altered if both acoustic and optical phonons are considered.

Another major source of decoherence is electrical noise. One can adopt a few strategies such as the following (besides biasing the system at the “sweet spot”) to reduce decoherence:

- Implementing a new geometry for a charge qubit, i.e., a linear triple quantum dot^41^. Unlike in a double quantum dot where fluctuations in uniform electric field (*ε*_*d*_) lead to decoherence, in a triple-dot geometry fluctuations of the field gradient (*ε*_*q*_) determine decoherence. The ratio ${\varepsilon }_{q}/{\varepsilon }_{d}\sim d/R$ where *d* is the separation between dots and *R* is the distance between dots and the remote source of charge noise such as a charge trap. In semiconductors *d*/*R* ~ 0.1 with *d* ~ 200 nm and *R* ~ 2000 nm; thus, there is an improvement for coherence times by at least an order of magnitude. Furthermore, for oxide-based dots, *d* can be at least an order of magnitude smaller. Thus the role of charge noise can be significantly diminished.
- One can also reduce the noise sensitivity through manipulating tunnel coupling by symmetrically lowering the potential barrier that separates the dots without changing the relative depth of the wells^42,43^.

However, given the less developed synthetic control and fabrication technology in oxides as compared to the situation in semiconductors, it is quite possible that electrical noise may produce much shorter decoherence times than optical phonons. Estimation of coherence times for charge noise requires additional extensive theoretical analysis of tunneling rates and detuning variations under charge fluctuation such as that reported in refs^44,45^.

Lastly, we will argue qualitatively that the nuclar spins, spin-spin interactions, and spin-orbit coupling will not have much effect on decoherence. When modeling manganite phenomena, coupling to nuclear spins is not important because hyperfine coupling is very small for d orbitals because only s orbitals have a finite amplitude at the nucleus; this situation is similar to holes in GaAs which occupy p orbitals^46^. Coupling to the core spins through Hund’s coupling is an important spin-spin interaction and produces ferromagnetism. In the ferromagnetic regime of *La*_1−*x*_*Ca*_*x*_*MnO*_3_ (at doping *x* = 0.3, Jahn-Teller polaronic energy *E*_*p*_ = 0.5 eV, *J*_⊥_ = 0.4 eV), the typical ferromagnetic interaction term^47^ is ~$x\mathrm{(1}-x){J}_{\perp }^{2}/\mathrm{(8}{E}_{p})=0.0084$ eV which is much larger than the thermal energy corresponding to the boiling point of liquid helium. Furthermore, using strong magnetic fields, the spin fluctuation effect on double-exchange-based tunneling can further be diminished. Thus spin-spin interaction will not significantly effect coherence. Unlike GaAs quantum dots^46^, since the g-factor is close to 2 in manganites^48^, the spin-orbit coupling in these perovskite oxides is negligible and spin relaxation due to spin-orbit coupling need not be considered.

In conclusion, our analysis (involving mapping a strong-coupling problem to a weak-coupling one) shows that oxides (such as manganites) may provide a useful material platform for realizing charge qubits with long coherence times at elevated temperatures (i.e., boiling point of liquid helium which is significantly above dilution refrigerator temperatures). However, experimental confirmation as well as further theoretical studies, involving sources of decoherence other than phonons, are needed to clearly establish that our manganite-based double quantum dot has an applicable combination of maneuverability and coherence time and that it clearly outperforms a semiconductor DQD. Thus, one needs advances beyond the interesting experimental studies on oxide-based single quantum dot reported in ref.^49^.

## Electronic supplementary material

Details of the calculation of matrix elements
