# Supplementary material for: Temperature dependence of long coherence times of oxide charge qubits
Source: Sci Rep. 2018 Feb 22;8:3487. doi: 10.1038/s41598-018-21767-2 (PMC5823872; doi:10.1038/s41598-018-21767-2)
Supplement: Supplementary file 1 — Details of the calculation of matrix elements [file 41598_2018_21767_MOESM1_ESM.pdf]

Temperature dependence of long coherence times of oxide charge qubits

A. Dey[1] and S. Yarlagadda[1]

[1]CMP Div., 1/AF Salt Lake, Saha Institute of Nuclear physics, Kolkata 700064, India.

Supplementary Information

Details of the calculation of matrix elements

Here we show the detailed calculations for the matrix elements obtained from the quantum master equation given by Eq. (5) in the main text. The double commutator in Eq. (5) can be broken into four terms. In the equation for the matrix element $\langle 10|\tilde{\rho}_s(t)|01\rangle$, the first term on the right-hand side [based on Eq. (5)] is given by

$$\begin{aligned}\langle 10|\sum_n {}_{ph}\langle n|\tilde{H}_I^L(t)\tilde{H}_I^L(\tau)|n\rangle_{ph}\tilde{\rho}_s(t)\frac{e^{-\beta\bar{\omega}_n}}{Z}|01\rangle \; = \; & \Big[\xi_{+,+}(\mathscr{T})\;\langle 10|\tilde{c}_1^+(t)\tilde{c}_2^-(t)\tilde{c}_1^+(\tau)\tilde{c}_2^-(\tau)\tilde{\rho}_s(t)|01\rangle \\ & +\xi_{+,-}(\mathscr{T})\;\langle 10|\tilde{c}_1^+(t)\tilde{c}_2^-(t)\tilde{c}_2^+(\tau)\tilde{c}_1^-(\tau)\tilde{\rho}_s(t)|01\rangle \\ & +\xi_{-,+}(\mathscr{T})\;\langle 10|\tilde{c}_2^+(t)\tilde{c}_1^-(t)\tilde{c}_1^+(\tau)\tilde{c}_2^-(\tau)\tilde{\rho}_s(t)|01\rangle \\ & +\xi_{-,-}(\mathscr{T})\;\langle 10|\tilde{c}_2^+(t)\tilde{c}_1^-(t)\tilde{c}_2^+(\tau)\tilde{c}_1^-(\tau)\tilde{\rho}_s(t)|01\rangle\Big].\end{aligned} \quad \text{(S1)}$$

Here, $\mathscr{T} = t - \tau$, i.e., the difference of times at which the the two-time correlation functions $\xi_{\mu,\nu}(\mathscr{T})$ are calculated. The correlation functions can be written as

$$\begin{aligned}&\xi_{\mu,\nu}(\mathscr{T}) \\ &= \frac{1}{4}\sum_{\{n_k\}} {}_{ph}\langle\{n_k\}|\tilde{J}_\perp^\mu(\mathscr{T})\tilde{J}_\perp^\nu|\{n_k\}\rangle_{ph}\frac{e^{-\beta\bar{\omega}_n}}{Z} \\ &= \frac{1}{4}\sum_{\{n_k\},\{m_k\}} {}_{ph}\langle\{n_k\}|\tilde{J}_\perp^\mu(\mathscr{T})|\{m_k\}\rangle_{ph}\; {}_{ph}\langle\{m_k\}|\tilde{J}_\perp^\nu|\{n_k\}\rangle_{ph}\frac{e^{-\beta\bar{\omega}_n}}{Z} \\ &= \frac{1}{4}\sum_{\{n_k\},\{m_k\}} {}_{ph}\langle\{n_k\}|J_\perp^\mu|\{m_k\}\rangle_{ph}\; {}_{ph}\langle\{m_k\}|J_\perp^\nu|\{n_k\}\rangle_{ph}e^{i(\bar{\omega}_n-\bar{\omega}_m)\mathscr{T}}\frac{e^{-\beta\bar{\omega}_n}}{Z},\end{aligned} \quad \text{(S2)}$$

where we observe that

$$\begin{aligned}&{}_{ph}\langle m_k|J_\perp^-|n_k\rangle_{ph} = {}_{ph}\langle n_k|J_\perp^+|m_k\rangle_{ph}, \\ &\text{and} \\ &{}_{ph}\langle m_k|J_\perp^+|n_k\rangle_{ph} = (-1)^{(n_1^k+n_2^k-m_1^k-m_2^k)}\,{}_{ph}\langle n_k|J_\perp^+|m_k\rangle_{ph}.\end{aligned} \quad \text{(S3)}$$

Now, we calculate the phonon correlation function $\xi_{+,+}(\mathscr{T})$ below:

$$\begin{aligned}&\xi_{+,+}(\mathscr{T}) \\ &= \frac{J_\perp^2}{4}\Bigg[\sum_{\{n_k\}} {}_{ph}\langle\{n_k\}|e^{-\frac{1}{\sqrt{N}}\sum_k g_k[\{a_{1,k}(\mathscr{T})-a_{1,k}^\dagger(\mathscr{T})\}-\{a_{2,k}(\mathscr{T})-a_{2,k}^\dagger(\mathscr{T})\}]}\times e^{-\frac{1}{\sqrt{N}}\sum_k g_k[\{a_{1,k}-a_{1,k}^\dagger\}-\{a_{2,k}-a_{2,k}^\dagger\}]}|\{n_k\}\rangle_{ph}\frac{e^{-\beta\bar{\omega}_n}}{Z} \\ &\quad -e^{-\frac{1}{N}\sum_k g_k^2\coth(\frac{\beta\omega_k}{2})}\sum_{\{n_k\}} {}_{ph}\langle\{n_k\}|e^{-\frac{1}{\sqrt{N}}\sum_k g_k[\{a_{1,k}-a_{1,k}^\dagger\}-\{a_{2,k}-a_{2,k}^\dagger\}]}|\{n_k\}\rangle_{ph}\frac{e^{-\beta\bar{\omega}_n}}{Z} \\ &\quad -e^{-\frac{1}{N}\sum_k g_k^2\coth(\frac{\beta\omega_k}{2})}\sum_{\{n_k\}} {}_{ph}\langle\{n_k\}|e^{-\frac{1}{\sqrt{N}}\sum_k g_k[\{a_{1,k}(\mathscr{T})-a_{1,k}^\dagger(\mathscr{T})\}-\{a_{2,k}(\mathscr{T})-a_{2,k}^\dagger(\mathscr{T})\}]}|\{n_k\}\rangle_{ph}\frac{e^{-\beta\bar{\omega}_n}}{Z} \\ &\quad +e^{-\frac{2}{N}\sum_k g_k^2\coth(\frac{\beta\omega_k}{2})}\sum_{\{n_k\}}\frac{e^{-\beta\bar{\omega}_n}}{Z}\Bigg]\end{aligned} \quad \text{(S4)}$$

The first term in Eq. (S4) is written as

$$\frac{J_\perp^2}{4}\sum_{\{n_k\}}\ {}_{ph}\langle\{n_k\}|e^{-\frac{1}{\sqrt{N}}\sum_k g_k[\{a_{1,k}(\mathscr{T})-a^\dagger_{1,k}(\mathscr{T})\}-\{a_{2,k}(\mathscr{T})-a^\dagger_{2,k}(\mathscr{T})\}]}\times e^{-\frac{1}{\sqrt{N}}\sum_k g_k[\{a_{1,k}-a^\dagger_{1,k}\}-\{a_{2,k}-a^\dagger_{2,k}\}]}|\{n_k\}\rangle_{ph}\frac{e^{-\beta\bar{\omega}_n}}{Z}$$

$$=\frac{J_\perp^2}{4}\sum_{\{n_1^k\}}\ {}_{ph}\langle\{n_1^k\}|e^{-\frac{1}{\sqrt{N}}\sum_k g_k\{a_{1,k}(\mathscr{T})-a^\dagger_{1,k}(\mathscr{T})\}}e^{-\frac{1}{\sqrt{N}}\sum_k g_k\{a_{1,k}-a^\dagger_{1,k}\}}|\{n_1^k\}\rangle_{ph}\frac{e^{-\beta\bar{\omega}_{n_1}}}{Z_1}$$

$$\times\sum_{\{n_2^k\}}\ {}_{ph}\langle\{n_2^k\}|e^{-\frac{1}{\sqrt{N}}\sum_k g_k\{a_{2,k}(\mathscr{T})-a^\dagger_{2,k}(\mathscr{T})\}}e^{-\frac{1}{\sqrt{N}}\sum_k g_k\{a_{2,k}-a^\dagger_{2,k}\}}|\{n_2^k\}\rangle_{ph}\frac{e^{-\beta\bar{\omega}_{n_2}}}{Z_2}$$

$$=\frac{J_\perp^2}{4}e^{-\frac{2}{N}\sum_k g_k^2\coth(\frac{\beta\omega_k}{2})[1+\cos(\omega_k\mathscr{T})]}e^{\frac{2}{N}i\sum_k g_k^2\sin(\omega_k\mathscr{T})}, \tag{S5}$$

where $Z_j=\sum_{\{n_j^k\}}e^{-\beta\bar{\omega}_{n_j}}$ and

$$\sum_{\{n_1^k\}}\ {}_{ph}\langle\{n_1^k\}|e^{-\frac{1}{\sqrt{N}}\sum_k g_k\{a_{1,k}(\mathscr{T})-a^\dagger_{1,k}(\mathscr{T})\}}e^{-\frac{1}{\sqrt{N}}\sum_k g_k\{a_{1,k}-a^\dagger_{1,k}\}}|\{n_1^k\}\rangle_{ph}\frac{e^{-\beta\bar{\omega}_{n_1}}}{Z_1}$$

$$=e^{-\frac{1}{N}\sum_k g_k^2}\sum_{\{n_1^k\}}\ {}_{ph}\langle\{n_1^k\}|e^{\frac{1}{\sqrt{N}}\sum_k g_k a^\dagger_{1,k}e^{i\omega_k\mathscr{T}}}e^{-\frac{1}{\sqrt{N}}\sum_k g_k a_{1,k}e^{-i\omega_k\mathscr{T}}}e^{\frac{1}{\sqrt{N}}\sum_k g_k a^\dagger_{1,k}}e^{-\frac{1}{\sqrt{N}}\sum_k g_k a_{1,k}}|\{n_1^k\}\rangle_{ph}\frac{e^{-\beta\bar{\omega}_{n_1}}}{Z_1}$$

$$=e^{-\frac{1}{N}\sum_k g_k^2(1+e^{i\omega_k\mathscr{T}})}\sum_{\{n_1^k\}}\ {}_{ph}\langle\{n_1^k\}|e^{\frac{1}{\sqrt{N}}\sum_k g_k a^\dagger_{1,k}(1+e^{i\omega_k\mathscr{T}})}e^{-\frac{1}{\sqrt{N}}\sum_k g_k a_{1,k}(1+e^{i\omega_k\mathscr{T}})}|\{n_1^k\}\rangle_{ph}\frac{e^{-\beta\bar{\omega}_{n_1}}}{Z_1}$$

$$=e^{-\frac{1}{N}\sum_k g_k^2\coth(\frac{\beta\omega_k}{2})(1+\cos\omega_k\mathscr{T})}e^{i\frac{1}{N}\sum_k g_k^2\sin\omega_k\mathscr{T}}. \tag{S6}$$

The second and third terms in Eq. (S4) are written as

$$e^{-\frac{1}{N}\sum_k g_k^2\coth(\frac{\beta\omega_k}{2})}\sum_{\{n_k\}}\ {}_{ph}\langle\{n_k\}|e^{-\frac{1}{\sqrt{N}}\sum_k g_k[\{a_{1,k}-a^\dagger_{1,k}\}-\{a_{2,k}-a^\dagger_{2,k}\}]}|\{n_k\}\rangle_{ph}\frac{e^{-\beta\bar{\omega}_n}}{Z}$$

$$=e^{-\frac{1}{N}\sum_k g_k^2\coth(\frac{\beta\omega_k}{2})}\sum_{\{n_k\}}\ {}_{ph}\langle\{n\}|e^{-\frac{1}{\sqrt{N}}\sum_k g_k[\{a_{1,k}(\mathscr{T})-a^\dagger_{1,k}(\mathscr{T})\}-\{a_{2,k}(\mathscr{T})-a^\dagger_{2,k}(\mathscr{T})\}]}|\{n_k\}\rangle_{ph}\frac{e^{-\beta\bar{\omega}_n}}{Z}$$

$$=\frac{J_\perp^2}{4}e^{-\frac{2}{N}\sum_k g_k^2\coth(\frac{\beta\omega_k}{2})}. \tag{S7}$$

The fourth term in Eq. (S4) is

$$\frac{J_\perp^2}{4}e^{-\frac{2}{N}\sum_k g_k^2\coth(\frac{\beta\omega_k}{2})}\sum_{\{n_k\}}\frac{e^{-\beta\bar{\omega}_n}}{Z}=\frac{J_\perp^2}{4}e^{-\frac{2}{N}\sum_k g_k^2\coth(\frac{\beta\omega_k}{2})}. \tag{S8}$$

Finally we get the simplified expression for $\xi_{+,+}(\mathscr{T})$

$$\xi_{+,+}(\mathscr{T})=\kappa^2\Big[e^{-\frac{2}{N}\sum_k g_k^2\coth(\frac{\beta\omega_k}{2})\cos(\omega_k\mathscr{T})}e^{\frac{2}{N}i\sum_k g_k^2\sin(\omega_k\mathscr{T})}-1\Big], \tag{S9}$$

where $\kappa=\frac{J_\perp^{\mathrm{mf}}}{2}$. In a similar fashion, the other correlation function can be written as

$$\xi_{+,-}(\mathscr{T})=\kappa^2\Big[e^{\frac{2}{N}\sum_k g_k^2\coth(\frac{\beta\omega_k}{2})\cos(\omega_k\mathscr{T})}e^{-\frac{2}{N}i\sum_k g_k^2\sin(\omega_k\mathscr{T})}-1\Big]. \tag{S10}$$

Using Eqs. (S9) and (S10) we write Eq. (S1) as

$$\langle 10|\sum_n {}_{ph}\langle n|\tilde{H}_I^L(t)\tilde{H}_I^L(\tau)|n\rangle_{ph}\tilde{\rho}_s(t)\frac{e^{-\beta\bar{\omega}_n}}{Z}|01\rangle$$

$$=\xi_{+,+}(\mathscr{T})\Big[-i\kappa Q(\mathscr{T})\big(P(t)P(\tau)+\kappa^2Q(\tau)Q(t)\big)\langle 01|\tilde{\rho}_s(t)|01\rangle+\kappa^2Q(\mathscr{T})\big(Q(t)P^*(\tau)-P(t)Q(\tau)\big)\langle 10|\tilde{\rho}_s(t)|01\rangle\Big]$$

$$+\xi_{+,-}(\mathscr{T})\Big[i\kappa\big(Q(t)P^*(\mathscr{T})P(\tau)-Q(\tau)P(t)P(\mathscr{T})\big)\langle 01|\tilde{\rho}_s(t)|01\rangle+\big(\kappa^2Q(t)Q(\tau)P^*(\mathscr{T})+P(t)P(\mathscr{T})P^*(\tau)\big)\langle 10|\tilde{\rho}_s(t)|01\rangle\Big]. \tag{S11}$$

The evolution of the system parts of the right-hand side of Eq. (S1) can be calculated using the following relations:

$$e^{-iH_s^L t}|10\rangle \quad = \quad [P(t)^*|10\rangle - i\kappa Q(t)|01\rangle]e^{i\frac{J_\parallel}{4}t}, \tag{S12}$$

and

$$e^{-iH_s^L t}|01\rangle \quad = \quad [P(t)|01\rangle - i\kappa Q(t)|10\rangle]e^{i\frac{J_\parallel}{4}t}, \tag{S13}$$

with $P(t) = \cos\Big(\mathrm{t}\sqrt{\frac{\Delta\varepsilon^2}{4}+\kappa^2}\Big) + \mathrm{i}\frac{\Delta\varepsilon}{2}\frac{\sin\Big(\mathrm{t}\sqrt{\frac{\Delta\varepsilon^2}{4}+\kappa^2}\Big)}{\sqrt{\frac{\Delta\varepsilon^2}{4}+\kappa^2}}$ and $Q(t) = \frac{\sin\Big(\mathrm{t}\sqrt{\frac{\Delta\varepsilon^2}{4}+\kappa^2}\Big)}{\sqrt{\frac{\Delta\varepsilon^2}{4}+\kappa^2}}$. For $\kappa \ll \Delta\varepsilon$, we can approximate Eq. (S11) as

$$\begin{aligned}\langle 10|\sum_n {}_{ph}\langle n|\tilde{H}_I^L(t)\tilde{H}_I^L(\tau)|n\rangle_{ph}\tilde{\rho}_s(t)\frac{e^{-\beta\bar{\omega}_n}}{Z}|01\rangle \quad &= \quad \xi_{+,-}(\mathscr{T})P(t)P(\mathscr{T})P^*(\tau)\langle 10|\tilde{\rho}_s(t)|01\rangle \\ &= \quad \xi_{+,-}(\mathscr{T})e^{i\Delta\varepsilon\mathscr{T}}\langle 10|\tilde{\rho}_s(t)|01\rangle.\end{aligned} \tag{S14}$$

The second term is given by

$$\begin{aligned}&\langle 10|\sum_n {}_{ph}\langle n|\tilde{H}_I^L(t)\tilde{\rho}_s(t)\otimes R_0\tilde{H}_I^L(\tau)|n\rangle_{ph}|01\rangle \\ &= \xi_{+,+}(-\mathscr{T})\Big[\langle 10|\tilde{\rho}_s(t)|10\rangle\big(-i\kappa P(t)P^2(\tau)Q(t) + i\kappa^3 Q(t)Q^2(\tau)P^*(t)\big) \\ &\quad +\langle 10|\tilde{\rho}_s(t)|01\rangle\big(\kappa^2 P(t)P(\tau)Q(t)Q(\tau) + \kappa^2 Q(t)Q(\tau)P^*(t)P^*(\tau)\big) \\ &\quad +\langle 01|\tilde{\rho}_s(t)|10\rangle\big(P^2(t)P^2(\tau) + \kappa^4 Q^2(t)Q^2(\tau)\big) \\ &\quad +\langle 01|\tilde{\rho}_s(t)|01\rangle\big(i\kappa P^2(t)P(\tau)Q(\tau) - i\kappa^3 Q^2(t)Q(\tau)P^*(\tau)\big)\Big] \\ &\ + \xi_{+,-}(-\mathscr{T})\Big[\langle 10|\tilde{\rho}_s(t)|10\rangle\big(-i\kappa^3 P(t)Q(t)Q^2(\tau) + i\kappa Q(t)P^*(t)P^2(\tau)\big) \\ &\quad +\langle 10|\tilde{\rho}_s(t)|01\rangle\big(-\kappa^2 P(t)P^*(\tau)Q(t)Q(\tau) - \kappa^2 Q(t)P^*(t)Q(\tau)P(\tau)\big) \\ &\quad +\langle 01|\tilde{\rho}_s(t)|10\rangle\big(\kappa^2 P^2(t)Q^2(\tau) + \kappa^2 Q^2(t)P^2(\tau)\big) \\ &\quad +\langle 01|\tilde{\rho}_s(t)|01\rangle\big(i\kappa^3 Q^2(t)Q(\tau)P(\tau) - i\kappa P^2(t)P^*(\tau)Q(\tau)\big)\Big].\end{aligned} \tag{S15}$$

For $\kappa \ll \Delta\varepsilon$, we get

$$\begin{aligned}\langle 10|\sum_n {}_{ph}\langle n|\tilde{H}_I^L(\tau)\tilde{\rho}_s(t)\otimes R_0\tilde{H}_I^L(t)|n\rangle_{ph}|01\rangle \quad &= \quad \xi_{+,+}(-\mathscr{T})\langle 01|\tilde{\rho}_s(t)|10\rangle P^2(t)P^2(\tau) \\ &= \quad \xi_{+,+}(-\mathscr{T})\langle 01|\tilde{\rho}_s(t)|10\rangle e^{i\Delta\varepsilon(t+\tau)}.\end{aligned} \tag{S16}$$

The third term is expressed as

$$\begin{aligned}&\langle 10|\sum_n {}_{ph}\langle n|\tilde{H}_I^L(\tau)\tilde{\rho}_s(t)\otimes R_0\tilde{H}_I^L(t)|n\rangle_{ph}|01\rangle \\ &= \xi_{+,+}(\mathscr{T})\Big[\langle 10|\tilde{\rho}_s(t)|10\rangle\big(-i\kappa P(\tau)P^2(t)Q(\tau) + i\kappa^3 Q(\tau)Q^2(t)P^*(\tau)\big) \\ &\quad +\langle 10|\tilde{\rho}_s(t)|01\rangle\big(\kappa^2 P(\tau)P(t)Q(t)Q(\tau) + \kappa^2 Q(\tau)Q(t)P^*(\tau)P^*(t)\big) \\ &\quad +\langle 01|\tilde{\rho}_s(t)|10\rangle\big(P^2(\tau)P^2(t) + \kappa^4 Q^2(\tau)Q^2(t)\big) \\ &\quad +\langle 01|\tilde{\rho}_s(t)|01\rangle\big(i\kappa P^2(\tau)P(t)Q(t) - i\kappa^3 Q^2(\tau)Q(t)P^*(t)\big)\Big] \\ &\ + \xi_{+,-}(\mathscr{T})\Big[\langle 10|\tilde{\rho}_s(t)|10\rangle\big(-i\kappa^3 P(\tau)Q(\tau)Q^2(t) + i\kappa Q(\tau)P^*(\tau)P^2(t)\big) \\ &\quad +\langle 10|\tilde{\rho}_s(t)|01\rangle\big(-\kappa^2 P(\tau)P^*(t)Q(\tau)Q(t) - \kappa^2 Q(\tau)P^*(\tau)Q(t)P(t)\big) \\ &\quad +\langle 01|\tilde{\rho}_s(t)|10\rangle\big(\kappa^2 P^2(\tau)Q^2(t) + \kappa^2 Q^2(\tau)P^2(t)\big) \\ &\quad +\langle 01|\tilde{\rho}_s(t)|01\rangle\big(i\kappa^3 Q^2(\tau)Q(t)P(t) - i\kappa P^2(\tau)P^*(t)Q(t)\big)\Big].\end{aligned} \tag{S17}$$

For $\kappa \ll \Delta\varepsilon$, we obtain

$$\begin{aligned}\langle 10|\sum_n {}_{ph}\langle n|\tilde{H}_I^L(\tau)\tilde{\rho}_s(t)\otimes R_0\tilde{H}_I^L(t)|n\rangle_{ph}|01\rangle &= \xi_{+,+}(\mathscr{T})\langle 01|\tilde{\rho}_s(t)|10\rangle P^2(\tau)P^2(t) \\ &= \xi_{+,+}(\mathscr{T})\langle 01|\tilde{\rho}_s(t)|10\rangle e^{i\Delta\varepsilon(t+\tau)}. \end{aligned} \tag{S18}$$

Lastly, the fourth term reads

$$\begin{aligned}&\langle 10|\sum_n \tilde{\rho}_s(t)\frac{e^{-\beta\bar{\omega}_n}}{Z}{}_{ph}\langle n|\tilde{H}_I^L(\tau)\tilde{H}_I^L(t)|n\rangle_{ph}|01\rangle \\ &= \xi_{+,+}(-\mathscr{T})\Big[i\kappa Q(\mathscr{T})\big(P(t)P(\tau)+\kappa^2 Q(\tau)Q(t)\big)\langle 10|\tilde{\rho}_s(t)|10\rangle + \kappa^2 Q(\mathscr{T})\big(Q(t)P^*(\tau)-P(t)Q(\tau)\big)\langle 10|\tilde{\rho}_s(t)|01\rangle\Big] \\ &\quad +\xi_{+,-}(-\mathscr{T})\Big[i\kappa(-Q(t)P^*(\mathscr{T})P(\tau)+Q(\tau)P(t)P(\mathscr{T}))\langle 10|\tilde{\rho}_s(t)|10\rangle + \big(\kappa^2 Q(t)Q(\tau)P^*(\mathscr{T})+P(t)P(\mathscr{T})P^*(\tau)\big)\langle 10|\tilde{\rho}_s(t)|01\rangle\Big].\end{aligned} \tag{S19}$$

For $\kappa \ll \Delta\varepsilon$, we can approximate the above expression as

$$\begin{aligned}\langle 10|\sum_n \tilde{\rho}_s(t)\frac{e^{-\beta\bar{\omega}_n}}{Z}{}_{ph}\langle n|\tilde{H}_I^L(\tau)\tilde{H}_I^L(t)|n\rangle_{ph}|01\rangle &= \xi_{+,-}(-\mathscr{T})P(t)P(\mathscr{T})P^*(\tau)\langle 10|\tilde{\rho}_s(t)|01\rangle \\ &= \xi_{+,-}(-\mathscr{T})e^{i\Delta\varepsilon\mathscr{T}}\langle 10|\tilde{\rho}_s(t)|01\rangle. \end{aligned} \tag{S20}$$

Putting Eqs. (S14), (S16), (S18), and (S20) in Eq. (5) of the main text and using the equalities in Eq. (S3), one obtains Eq. (8) in the main text. In a similar way, one can deduce Eq. (9) for the diagonal element. Eqs. (6) and (7) can be deduced by using Eqs. (S11), (S15), (S17), and (S19) without the approximation $\kappa \ll \Delta\varepsilon$ and re-expressing the terms in $\{|\varepsilon_s\rangle, |\varepsilon_t\rangle\}$ basis. It should be noted that, at zero detuning, the only system excitation is given by $\delta\varepsilon = 2\kappa = J_\perp^{\mathrm{mf}}$.
